# Extensive Translational Regulation through the Proliferative Transition of Trypanosoma cruzi Revealed by Multi-Omics

**DOI:** 10.1128/mSphere.00366-21

**Published:** 2021-09-01

**Authors:** Santiago Chávez, Michael D. Urbaniak, Corinna Benz, Pablo Smircich, Beatriz Garat, José R. Sotelo-Silveira, María Ana Duhagon

**Affiliations:** a Laboratorio de Interacciones Moleculares, Facultad de Ciencias, Universidad de la República, Montevideo, Uruguay; b Departamento de Genética, Facultad de Medicina, Universidad de la República, Montevideo, Uruguay; c Departamento de Genómica, Instituto de Investigaciones Biológicas Clemente Estable, Ministerio de Educación y Cultura, Montevideo, Uruguay; d Biomedical and Life Sciences, Faculty of Health and Medicine, Lancaster University, Lancaster, United Kingdom; e Departamento de Biología Celular y Molecular, Facultad de Ciencias, Universidad de la República, Montevideo, Uruguay; University of Texas Southwestern

**Keywords:** Chagas’ disease, *Trypanosoma*, *Trypanosoma cruzi*, cell cycle, cell proliferation, genomics, mass spectrometry, posttranscription, proteomics, regulon, ribosome profiling, translational control

## Abstract

Trypanosoma cruzi is the etiological agent for Chagas disease, a neglected parasitic disease in Latin America. Gene transcription control governs the eukaryotic cell replication but is absent in trypanosomatids; thus, it must be replaced by posttranscriptional regulatory events. We investigated the entrance into the T. cruzi replicative cycle using ribosome profiling and proteomics on G_1_/S epimastigote cultures synchronized with hydroxyurea. We identified 1,784 translationally regulated genes (change > 2, false-discovery rate [FDR] < 0.05) and 653 differentially expressed proteins (change > 1.5, FDR < 0.05), respectively. A major translational remodeling accompanied by an extensive proteome change is found, while the transcriptome remains largely unperturbed at the replicative entrance of the cell cycle. The differentially expressed genes comprise specific cell cycle processes, confirming previous findings while revealing candidate cell cycle regulators that undergo previously unnoticed translational regulation. Clusters of genes showing a coordinated regulation at translation and protein abundance share related biological functions such as cytoskeleton organization and mitochondrial metabolism; thus, they may represent posttranscriptional regulons. The translatome and proteome of the coregulated clusters change in both coupled and uncoupled directions, suggesting that complex cross talk between the two processes is required to achieve adequate protein levels of different regulons. This is the first simultaneous assessment of the transcriptome, translatome, and proteome of trypanosomatids, which represent a paradigm for the absence of transcriptional control. The findings suggest that gene expression chronology along the T. cruzi cell cycle is controlled mainly by translatome and proteome changes coordinated using different mechanisms for specific gene groups.

**IMPORTANCE** Trypanosoma cruzi is an ancient eukaryotic unicellular parasite causing Chagas disease, a potentially life-threatening illness that affects 6 to 7 million people, mostly in Latin America. The antiparasitic treatments for the disease have incomplete efficacy and adverse reactions; thus, improved drugs are needed. We study the mechanisms governing the replication of the parasite, aiming to find differences with the human host, valuable for the development of parasite-specific antiproliferative drugs. Transcriptional regulation is essential for replication in most eukaryotes, but in trypanosomatids, it must be replaced by subsequent gene regulation steps since they lack transcription initiation control. We identified the genome-wide remodeling of mRNA translation and protein abundance during the entrance to the replicative phase of the cell cycle. We found that translation is strongly regulated, causing variation in protein levels of specific cell cycle processes, representing the first simultaneous study of the translatome and proteome in trypanosomatids.

## INTRODUCTION

Trypanosoma cruzi is the etiologic agent of Chagas disease, a lifelong debilitating illness that affects approximately 7 million people mainly in Latin America, causing 10,000 annual deaths (1). This insect-borne disease is a complex zoonosis that extends to a wide range of mammals and is transmitted by hundreds of blood-sucking triatomine bugs in areas of endemicity (2). Currently, there are no effective treatments or vaccines, and several authors have highlighted the need to better understand parasite replication as a target for drug development (3).

In eukaryotes, the highly coordinated sequence of events occurring in a unidirectional manner to ensure the generation of two new daughter cells is known as the cell cycle. There is an elaborate temporal control of gene expression along the cell cycle, directed by checkpoints and molecular regulators that dictate its progression. For most of the eukaryotic models, this periodical gene expression is strongly dependent on transcriptional control, where networks of transcription factors act to regulate the expression of large sets of mRNAs (4–7). In addition, targeted protein degradation at specific times of the cell cycle is a key step to ensure progression to subsequent phases (8); thus, proteomic analyses are of major relevance to understand the cell cycle (9). Nevertheless, the translational regulation of mRNAs, which represent a main determinant of protein abundance, has not been studied in a genome-wide fashion during the cell cycle until recently. Ribosome profiling consists of the deep sequencing of ribosome-protected mRNA fragments (footprints) and has proved to allow a highly accurate measurement of the translation process on a genome-wide basis (10, 11). The development of this approach led to the first reports of translatome remodeling on the human (12, 13) and budding yeast (14) cell cycle.

As a single-celled organism, T. cruzi continuously adapts to changing environments and alternates between replicating forms (epimastigotes and amastigotes) and quiescent infective forms (trypomastigotes), thus needing to rapidly alter gene expression programs (15, 16). Transcription by RNA polymerase II is constitutive and polycistronic in T. cruzi, producing continuous RNAs with tens to hundreds of functionally unrelated protein-coding genes. These primary transcripts are processed by 5′ *trans*-splicing and 3′ polyadenylation to form mature canonical mRNA molecules. Given these unusual gene expression mechanisms, T. cruzi mostly relies on the posttranscriptional levels of regulation, such as control of mRNA localization, stability, and translational efficiency to achieve differential protein synthesis (17–23).

The cell cycle of T. cruzi presents particularities such as the coordination of the nuclear division with highly polarized cell structures like the single-copy organelles, including the basal body, flagellum, endoplasmic reticulum (ER), Golgi complex, and the large single mitochondrion with a genome composed of multiple circular DNA molecules known as the kinetoplast (24, 25). In fact, it has been established that the mitochondrial and the nuclear genomes undergo separate S phases and that their segregation is under temporal control by the interaction with distinct microtubule-based structures like the basal bodies and the mitotic spindle (26). In addition, trypanosomatids display a closed mitosis with no chromosome condensation (27) and unique divergent kinetochores components and an unclear spindle checkpoint (28, 29). Cyclins and cyclin-dependent kinases (CDKs) are conserved in trypanosomatids (30–33), so the. mechanisms that govern cell cycle progression are likely to be similar to those of higher eukaryotes. The eukaryotic cell cycle is strongly controlled by gene transcription, including cyclin periodical expression and transcription factor programs needed for its progression. However, T. cruzi lacks transcriptional control; thus, the molecular mechanism governing proliferation must be divergent from those described in model organisms (34, 35). Overall, divergence in the cell biology and gene expression control (36) between the parasite and human host proliferative cycle turns it into a focus for drug development efforts (37–39). In trypanosomatids, RNA binding proteins (RBPs) have been proposed as surrogates for transcription factors for the coordinated regulation of groups of mRNAs, a regulatory network known as posttranscriptional regulons (40–42), which may also underlie the control of cell cycle progression in T. cruzi. Yet, little is known about the regulons driving the periodical gene expression in trypanosomatids (43), while only one related study has been published on T. cruzi so far (44).

Recently, genome-wide approaches have been carried out using synchronic Trypanosoma brucei parasite populations to generate cell cycle profiles at the transcriptomic (45) and proteomic and phosphoproteomic (46) levels. A similar transcriptomic study was published by our group on T. cruzi (44), revealing that the steady-state mRNA abundance is regulated as the cycle progresses, although only a small number of genes (305) showed statistically significant changes. However, genome-wide multilevel comparisons of the transcriptome, translatome, and proteome during the cell cycle are currently scarce in the literature and have not been performed in trypanosomatids. The relevance of posttranscriptional regulation of gene expression in trypanosomatids turns them into a particularly interesting model for multi-omic studies.

Here, we provide the first ribosome profiling study of the trypanosomatid cell cycle, and in parallel, we determined the quantitative proteome. The global patterns of gene expression regulation were compared at three levels during the proliferative transition (G_1_/S) of synchronized epimastigote populations. We determined the differentially expressed genes (DEGs), identifying their specific levels of control. The ontological gene terms enriched in each phase highlight known cell cycle pathways and novel periodically expressed proteins. Cell cycle regulators, including cyclins, CDKs, and RBPs differentially expressed during the G_1_/S transition, are analyzed. Our results show the outstanding role of translational regulation in the two cell cycle phases studied. Coexpressed groups of functionally related genes that may comprise regulons were identified. The comparison of the regulatory levels reveals a complex and regulon-specific interplay between the translatome and the proteome. Our study improves the understanding of T. cruzi proliferation and raises novel hypotheses about the multilevel regulation of gene expression in the cell cycle.

## RESULTS

### Translational and proteomic remodeling during T. cruzi epimastigote G_1_/S cell cycle transition.

**FIG 1:**
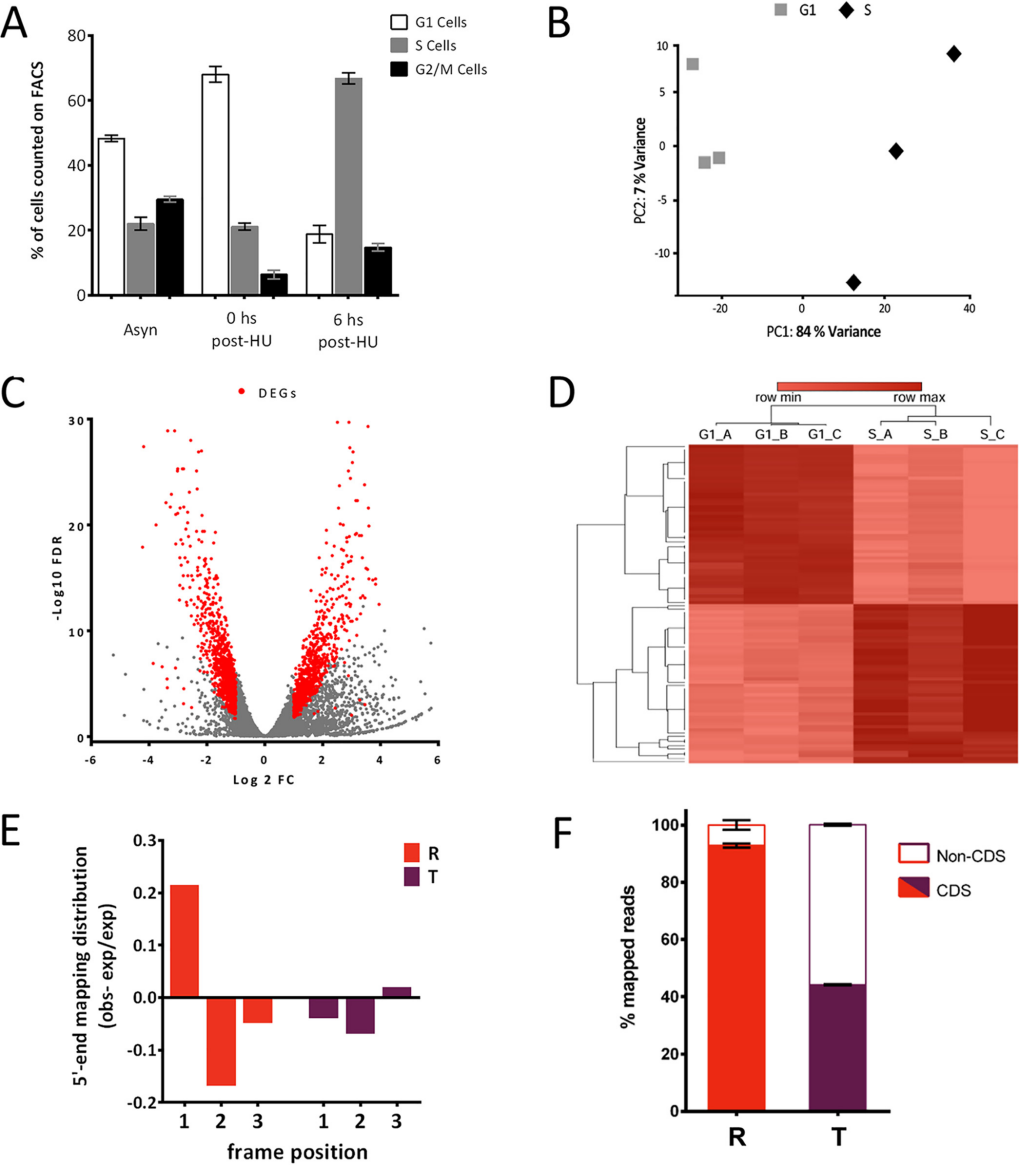
Ribosome profiling data set. (A) A fluorescence-activated cell sorting analysis of DNA content staining with propidium iodide was carried out for HU-synchronized T. cruzi populations. The distributions of G_1_ (2C cells, white), S (2-4C cells, gray), and G_2_/M (4C cells, black) are presented for an asynchronous parasite culture (Asyn), a G_1_-enriched population (0 h post-HU), and an S-phase-enriched population (6 h post-HU). (B) Principal-component analysis of the gene expression values for the replicates of G_1_-enriched population (gray squares) and S-enriched population (black diamonds). (C) Volcano plot showing the distribution of FDR values for each gene versus the fold change in expression. Red dots are genes classified as DEGs (FC > 2, FDR < 0.05, nRFPs > 40). (D) Heatmap representing the 50 up- and downregulated genes (log_2_ nRFP values are presented). (E) Observed (obs)-to-expected (exp) ratio of the 5′-end footprint mapping distribution in the three reading frames, analyzed for the ribosome profiling (R) and the transcriptome (T). (F) Average mapping position was taken and counted among coding (CDS, dark gray) and noncoding (non-CDS, light gray) sequences for the ribosome profiling (R) and the transcriptome (T).

Hydroxyurea (HU)-induced synchronization was used to obtain cell cycle-enriched populations of T. cruzi epimastigotes (44, 47), achieving enrichments of approximately 70% for each of the desired populations (Fig. 1A). After incubation with HU, cultures were harvested at 0 h (G_1_) and 6 h (S) and used for polysome isolation. Ribosome footprints were prepared by digestion of polyribosomal fractions obtained by ultracentrifugation on sucrose cushions, as conducted previously (23). The resulting ribosome-protected fragments, also known as ribosome footprints, were used to generate high-throughput sequencing libraries. Read counts from the libraries are a measure of ribosome occupancy on the mRNAs, thus a surrogate measurement of protein translation. Although this approach cannot distinguish active and stalled ribosomes, it has emerged as a comprehensive and quantitative method to assess translation at a genome-wide level (10). Samples were sequenced on a Novaseq 6000 (Illumina) producing over 18 and 13 million 76-bp reads for G_1_ and S, respectively, mapped to the T. cruzi CL-Brener Esmeraldo-like haplotype genome (see Table S1 in the supplemental material). To isolate reads resulting from ribosome-protected mRNA fragments, Illumina’s 3′-adapter was identified and only the trimmed reads were retained for mapping. Read counts per gene were calculated and normalized, resulting in a translation estimate (normalized ribosome footprints [nRFPs]) presented for 9,487 genes with detectable translation (Table S2). The triplicates displayed an expected distribution on the principal-component analysis, where the replicates were clustered together and separated from each other by a first component that contains 84% of the variance (Fig. 1B). Differential translation analysis was carried out, and genes with more than 40 nRFPs and showing a fold change of >2 and a false-discovery rate (FDR) of <0.05 were considered differentially expressed genes at the translational level (R-DEGs; see Materials and Methods for further information). This resulted in 1,784 G_1_/S R-DEGs, with 923 and 861 G_1_ and S upregulated genes, respectively (Fig. 1C and D; Table S2).

10.1128/mSphere.00366-21.7 TABLE S1 Omics throughput data. Download Table S1, XLSX file, 0.01 MB. Copyright © 2021 Chávez et al. 2021 Chávez et al. https://creativecommons.org/licenses/by/4.0/ This content is distributed under the terms of the Creative Commons Attribution 4.0 International license.

10.1128/mSphere.00366-21.8 TABLE S2 Gene expression analysis for the three data sets. Download Table S2, XLSX file, 7.1 MB. Copyright © 2021 Chávez et al. 2021 Chávez et al. https://creativecommons.org/licenses/by/4.0/ This content is distributed under the terms of the Creative Commons Attribution 4.0 International license.

As a quality control, read mappings resulting from a ribosome footprint assay should display a 5′-end 3-bp mapping periodicity and should map preferably over protein coding sequences (10, 23). For the first analysis, we discriminated the reads by length, determined the p-site offset for each read length, and calculated the 5′ periodicity taking genes displaying a continuous overall coverage as previously reported (10). Periodicity was observed in the translatome data as the RFPs mapped more frequently to the first codon position, and the second codon position was the least represented (11). As expected, the transcriptome data did not display this 3-nucleotide (nt) periodicity (Fig. 1E). Ribosome footprints predominantly map to protein coding regions as 93% of the reads fall within the initiation and stop codons as expected. This was not the case for polyadenylated reads coming from the transcriptome study, since only 45% of these reads mapped to coding regions while the remaining mapped to the untranslated regions (UTRs) and intergenic regions (Fig. 1F). Both observations support that RFP reads correspond to transcripts actively translated by polyribosomes.

**FIG 2:**
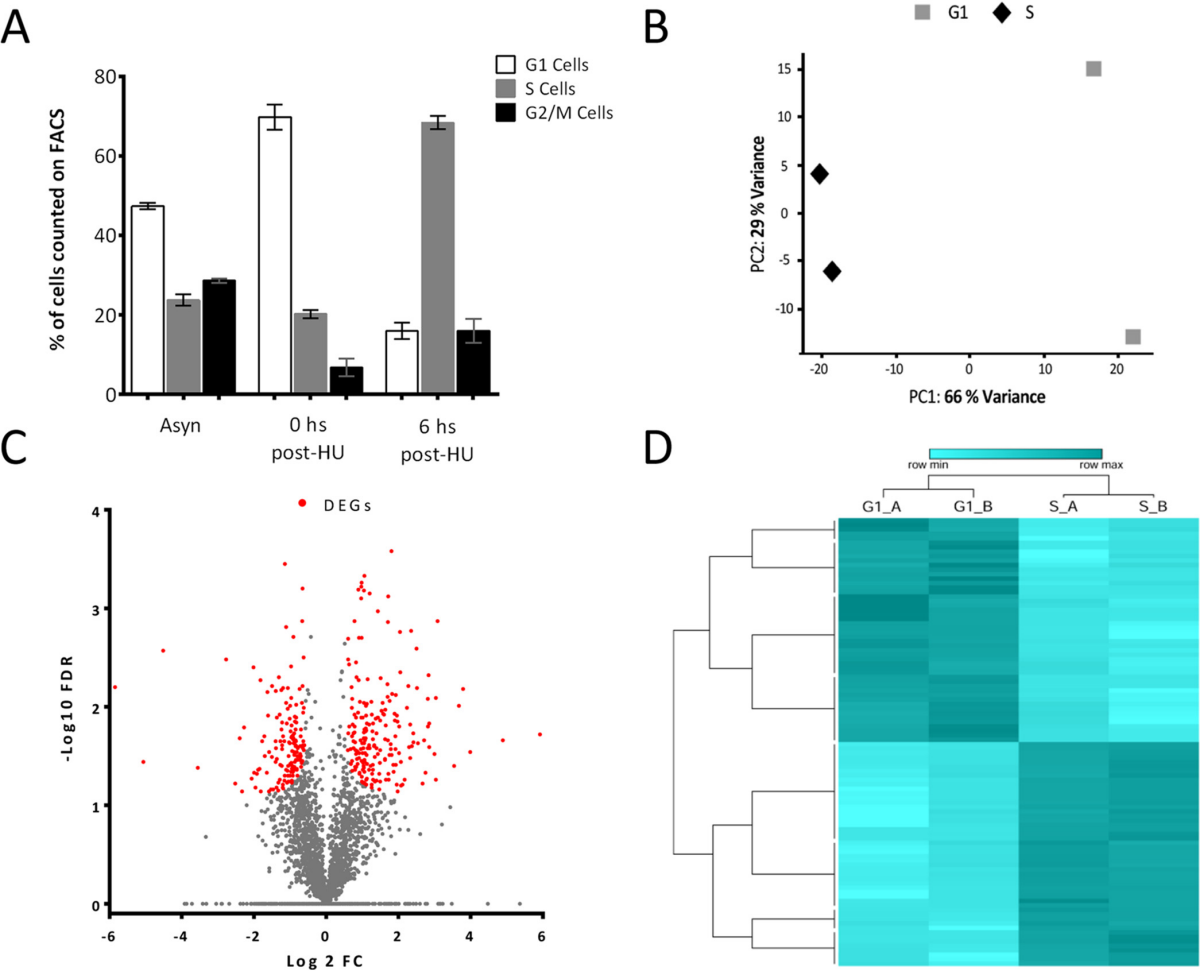
Proteomic data set. (A) A fluorescence-activated cell sorting analysis of DNA content staining with propidium iodide was carried out for HU-synchronized T. cruzi populations. The distributions of G_1_ (2C cells, white), S (2-4C cells, gray), and G2/M (4C cells, black) are presented for an asynchronous parasite culture (Asyn), a G_1_-enriched population (0 h post-HU), and an S-phase-enriched population (6 h post-HU). (B) Principal-component analysis of the LFQ values for the duplicates of G_1_-enriched population (gray squares) and S-enriched population (black diamonds). (C) Volcano plot showing the distribution of FDR values for each gene versus the fold change in expression. Red dots are genes classified as DEGs. (D) Heatmap representing the top 50 up- and downregulated genes (transformed log_2_ LFQ values are presented).

Protein extracts of the G_1_ (0 h post-HU release) and S-phase (6 h post-HU release) enriched cell cycle populations were obtained with a protocol identical to that used for the preparation of ribosome footprints (Fig. 2A). Tryptic peptide-digested samples were processed in parallel using high-pH reversed-phase fractionation to reduce complexity prior to analysis by liquid chromatography-tandem mass spectrometry (LC-MS/MS) (Table S1). Principal-component analysis showed a correct grouping of the replicates, demonstrating separation of the data sets by the first component that contained 66% of the variance (Fig. 2B). Data analysis identified 4,524 protein groups containing 4,918 proteins, with normalized label-free quantification (LFQ) intensity values presented in Table S2. The lower sensitivity and range of the proteomic detection, compared to the Ribo-seq, together with the use of only two replicates, which increases the likelihood of type II errors, led us to use a 1.5-fold change for differential protein expression assessment. We considered only protein groups supported by an FDR lower than 0.05. Following these criteria, 408 genes (8.3% of total) displayed altered protein levels, with 168 and 240 upregulated genes in G_1_ and S, respectively (Fig. 2C and D). We also incorporated the proteins detected in both replicates of one cell cycle stage (above the 5th percentile in LFQ values) but absent from the other, resulting in an additional 36 and 208 proteins for G_1_ and S, respectively. Therefore, in total we identified 652 genes regulated at the protein abundance level (P-DEGs) in the G_1_/S transition, of which 204 and 448 are upregulated at G_1_ and S phase, respectively.

### Comparison of the transcriptomic, translatomic, and proteomic changes at the T. cruzi epimastigote G_1_/S cell cycle transition.

We have generated transcriptomic (previous study [44]) and translatomic and proteomic (current study) data sets of G_1_/S cell cycle phases of T. cruzi epimastigotes of the same TcI strain. For all studies, an identical protocol was used to handle the samples and a highly reproducible cell cycle distribution of the cultures was obtained.

**FIG 3:**
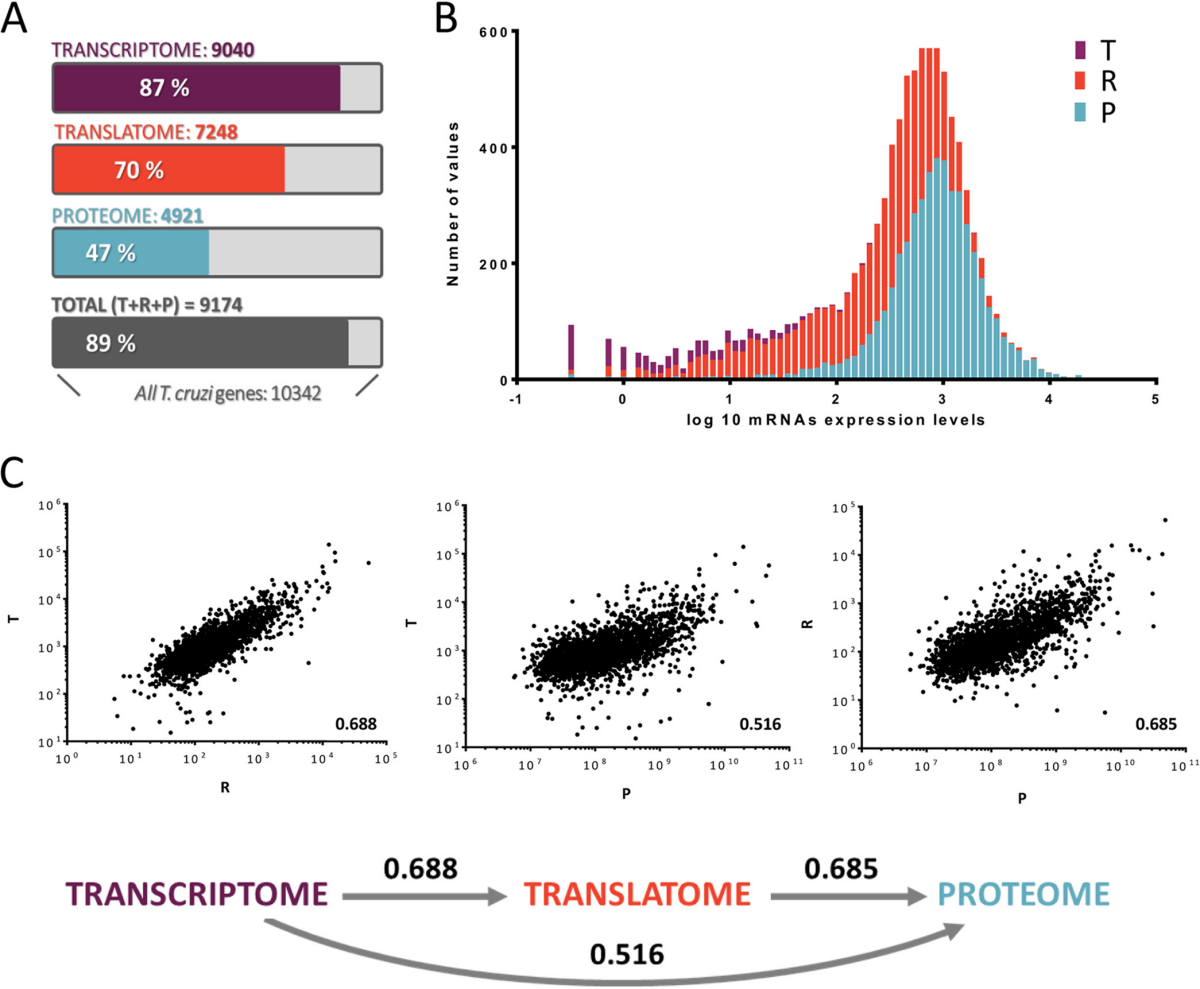
Data set comparisons. (A) The threshold for detection was 15 nCounts for both the transcriptome (T, violet) and translatome (R, red), and at least 1 unique peptide for the proteome (P, blue). (B.) The histogram presents the mRNA levels for the set of detected genes in each data set. (C) Dot plots for pairwise gene expression comparisons of the two data sets. R values for Pearson correlations for the comparisons are presented above the arrows connecting the data sets analyzed.

In order to compare the sensitivities of the three approaches used, we set a detection threshold of 15 raw reads per gene in the sequencing studies (23) and 1 unique peptide for the proteomic study (46) (Fig. 3A). We had previously identified 9,040 genes of the 10,342 T. cruzi protein coding genes in the epimastigote transcriptome (44). In the current study, the ribosome profiling revealed 7,248 genes with detectable levels of translation, which represent 70% of the T. cruzi coding genes and 80% (7,242 of 9,040 genes) of the transcribed mRNAs (Fig. S1). This large proportion of translated transcripts has been previously reported in T. cruzi epimastigotes (23). Interestingly, the most enriched term among the 1,600 transcripts detected in the transcriptome but not in the translatome is “pseudogene” (33% of the genes) followed by surface protein-related terms (Fig. S1). Moreover, we compared this gene list with the set of 526 genes present only in the metacyclic trypomastigote translatome as published by Smircich and collaborators (23) and found a significant coincidence of these genes (Fig. S1). Both observations represent a validation of biological relevance for the transcriptome and translatome data sets, as genes related to the infective stages are transcribed but are not selected for translation in the epimastigote stage. Finally, 4,524 protein groups were detected at the proteomic level, which corresponds to 4,918 genes, i.e., 47%, representing a high coverage of the genome in comparison to previous studies (48, 49) (Fig. 3A). As anticipated by the lower sensitivity of the shotgun proteomics in comparison to transcriptome sequencing (RNA-seq), this gene group is biased toward high-abundance transcripts (Fig. 3B); yet, they represent 67% (4,918 of 7,248) of the translated transcripts (Fig. S1). Pairwise comparisons of G_1_/S averaged values for the three data sets show Pearson correlation values larger than 0.5, indicating an agreement in the gene expression levels measured by all three different experiments (Fig. 3C). As previously reported, the translatome proved to be a better proxy for protein levels than the transcriptome (0.685 versus 0.516) (11). During the G_1_/S transition the translatome and proteome display a broader regulation than the transcriptome, demonstrated by a higher number and proportion of DEGs relative to analyzed genes (transcriptome: 305/9,087, 3%; translatome: 1,784/7,530, 24%; proteome: 653/4,254, 16%) (Fig. S2). Likewise, a wider dynamic range of variation (expressed as median log_2_ fold change) supports the broader regulation observed for the translatome (0.66) and proteome (0.48) data sets than for the transcriptome (0.16) (Fig. S2).

10.1128/mSphere.00366-21.2 FIG S1 Gene expression detection for each data set. (A) Venn diagram displaying the intersection of genes detected in each data set. For the sequencing studies (transcriptome [T] and translatome [R]), 15 normalized reads were taken as the only cutoff, while for the proteome (P), all proteins displaying 1 unique peptide were considered. (B) A word enrichment analysis was performed in TriTrypDB for product description of the 1,600 genes detected in the T but not in the R or the P. The top 10 nonredundant enriched terms are presented in a word cloud made using WocEA software (W. Ning, S. Lin, J. Zhou, Y. Guo, et al., J Genet Genomics 45:415–417, 2018, https://doi.org/10.1016/j.jgg.2018.02.008). Word size represents fold enrichment, and color represents significance of Bonferroni-adjusted *P* values (high = 6.9E−148, low = 6.2E−3). (C) Venn diagram presents the intersection of 1,600 genes detected in the T but not in the R or the P with the list of 526 genes only detected in the metacyclic trypomastigote translatome by Smircich et al. (23). The resulting 253 genes present in the intersection represent a significant enrichment as assessed by chi-square with Yates correction test with a *P* value of <0.0001. The gene list used in these analyses can be retrieved by filtering Table S2 for genes with more than 15 reads in the T data, fewer than 15 reads in the R data, and no data in the P. Download FIG S1, TIF file, 0.9 MB. Copyright © 2021 Chávez et al. 2021 Chávez et al. https://creativecommons.org/licenses/by/4.0/ This content is distributed under the terms of the Creative Commons Attribution 4.0 International license.

10.1128/mSphere.00366-21.3 FIG S2 Comparison of the S/G_1_ changes observed in the three data sets. (A) Graphical representation of the proportion of genes classified as DEGs in each data set compared to the total genes analyzed for differential expression. (B) Dynamic range of the changes in the transcriptome, translatome, and proteome (T-R-P) data sets. Absolute change between G_1_ and S phase is presented for all genes with measurable fold change values in each data set. The Mann-Whitney test for non-Gaussian distributed data was performed to compare each data set’s mean values. Significance was determined with a *P* value lower than 0.05 (**** is <0.0001). DE, differential expression. Download FIG S2, TIF file, 0.1 MB. Copyright © 2021 Chávez et al. 2021 Chávez et al. https://creativecommons.org/licenses/by/4.0/ This content is distributed under the terms of the Creative Commons Attribution 4.0 International license.

**FIG 4:**
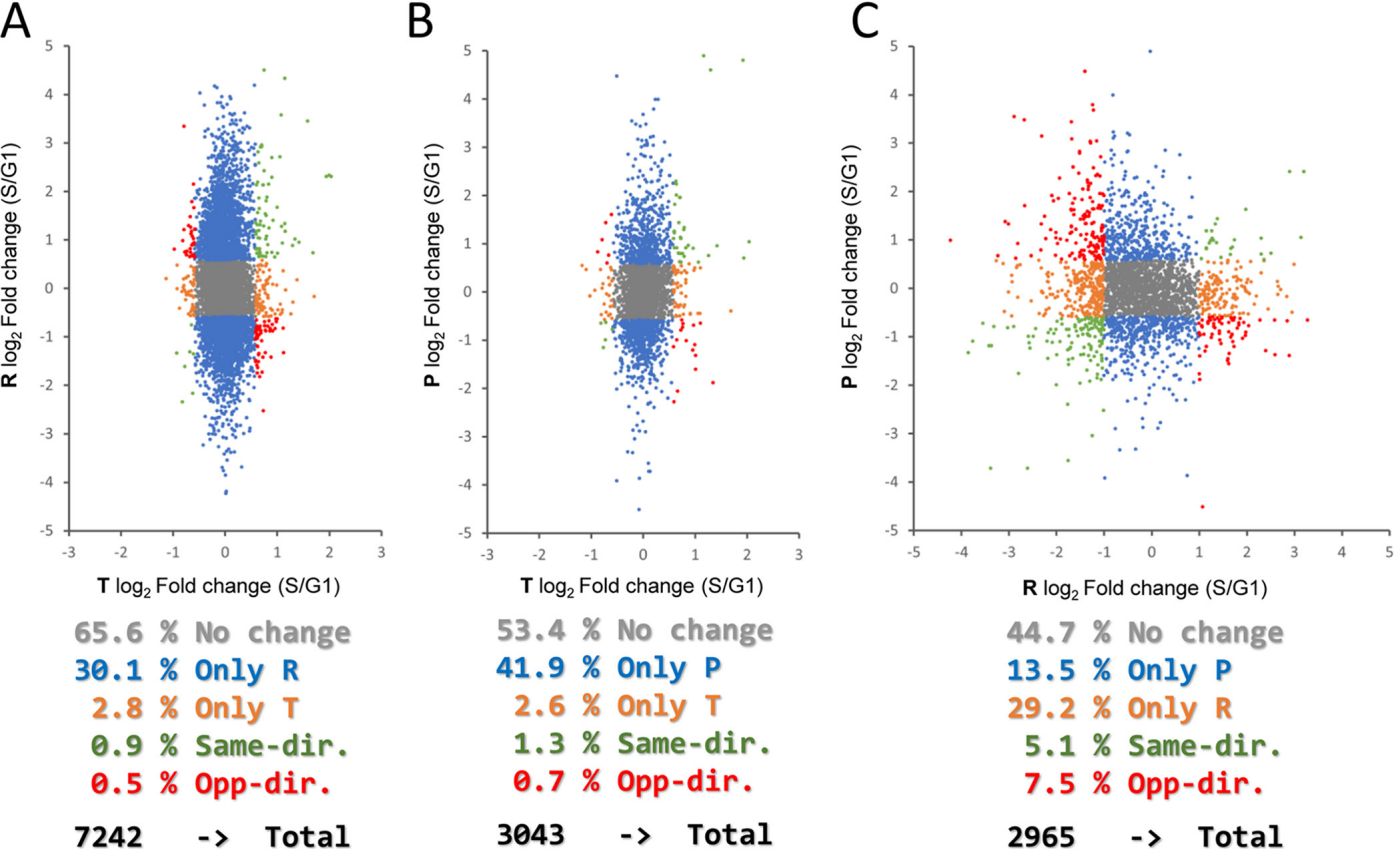
Correlations of S/G_1_ changes. Log_2_ fold change comparisons between the 3 data sets. Transcriptome data set is from the work of Chavez et al., 2017 (44). For this analysis, we included genes with 15 normalized reads for the sequencing studies (transcriptome [T] and translatome [R]) and at least 1 valid LFQ value for one replicate of each of the two cell cycle phases studied for the proteome (P). The number of genes analyzed in each diagram resulting from the threshold mentioned above is presented at the bottom. Fold changes were taken from average gene expression for the replicates for both R and P. Genes displaying changes are colored as described below the plots; the thresholds considered are those used to define DEGs in further analysis (1.5-fold for T and P and 2-fold for R). (A) Translatome versus transcriptome. (B) Proteome versus transcriptome. (C) Translatome versus proteome.

A broad picture of the expression changes observed in the G_1_/S transition at the three different levels of gene expression analyzed is presented in Fig. 4. For this initial analysis we included a larger proportion of the data sets, comprising genes with 15 normalized reads for the sequencing studies and at least 1 valid LFQ value in one replicate for each cycle phase for the protein quantification analysis. Globally, the changes observed at the translatome are larger in number and dynamic range than those observed at the transcriptome (Fig. 4A), confirming that T. cruzi epimastigotes rely on posttranscriptional regulation to achieve differential gene expression in the G_1_/S cell cycle transition. Additionally, over one-third of the genes plotted in Fig. 4A change in either the translatome or the transcriptome, while 30% do so only at translation and 3% only at mRNA abundance. As expected, most of the genes modulated at both the translatome and the transcriptome exhibit changes in the same direction, while a small fraction of them (36 genes) display changes in opposite directions. It is worth noting that in the context of an exceptionally small modulation of transcript abundance, changes in translational efficiency are almost equivalent to the changes in ribosome occupancy. Therefore, we will not apply the normalization by transcriptomic changes on the translatome data and we will use the ribosome density to describe translational variations from here on.

As expected, the comparison between the transcriptome and the proteome also shows that proteomic variations are more numerous and larger than those of transcriptomics (Fig. 4B). Yet, due to proteomic sensitivity limitations, fewer genes are included in this analysis (3,043) relative to the transcriptome-translatome comparison. Over half of these genes (53%) do not change their levels, neither in the proteome nor in the transcriptome. The vast majority of the modulated genes show changes only in the proteome (41%), while few of them change only in the transcriptome (2%), reinforcing the relevance of posttranscriptional mechanisms to achieve differential protein levels from similarly abundant mRNAs. From the subset of genes that displayed changes in both studies (62), again most of these genes (40 genes representing 1.3% of the total) showed changes in the same direction.

The fraction of regulated genes identified in the translatome-proteome comparison is the highest among the three data sets (55% of the genes, translatome fold change > 2 and proteome fold change > 1.5) (Fig. 4C). While 41.8% exhibit differential levels in the proteome and 26.1% in the translatome study, only 12.6% of them present differential levels in both studies. If the latter observation holds true for the genes not detected at protein level, it will indicate that the regulation of protein stability affects more genes than the translational control of mRNAs during the G_1_/S cell cycle transition. Since the coordination of protein modifications and stability represents a molecular hallmark of the cell cycle transitions (50), being essential for the G_1_/S transition (85), a strong regulation of protein levels is expected. Unexpectedly, changes in opposite directions (222 out of 372, 7%) are more frequent than those in the same direction (150, 5%). The uncoupling of gene expression changes in omics experiments has been discussed recently and proved to be more common than previously recognized (51). As presented above, differences in the magnitude of modulation in each data set could contribute to the profiles observed (Fig. S2). An alternative explanation is possible if protein half-life is longer than the polysomal mRNA activity, and so variations in production in the translatome have less effect on the proteome. This hypothesis will be addressed below in this work.

### Genes and biological process differentially regulated at the translatome and proteome during T. cruzi epimastigote G_1_/S cell cycle transition.

Seeking to investigate the biological function of the DEGs along the T. cruzi G_1_/S transition, we searched for enriched gene ontology terms (Table 1; complete list of terms and genes in Table S3). For this study we used the R-DEGs and P-DEGs, i.e., those genes significantly regulated at a threshold of 2-fold for translatome and 1.5-fold for proteome (FDR of 0.05 for both studies). We added the previously identified transcriptomic DEGs (T-DEGs) for comparison reasons (44). Ontological analysis in trypanosomatids should be interpreted with caution given that annotations of their genomes are less precise than in model organisms.

**TABLE 1:** Enriched Gene Ontology terms of G_1_/S DEGs^*a*^

G_1_-enriched GO terms				S-enriched GO terms			
Name	No. of genes	FE	*P*-val	Name	No. of genes	FE	*P*-val
**T-DEGs**							
Monosaccharide binding (MF)	3	56.6	1.8E−03	DNA binding (MF)	13	6.0	3.0E−05
Ligase activity, forming carbon-nitrogen bonds (MF)	4	17.4	9.8E−03	***DNA replication (BP)***	8	11.6	1.4E−04
Nucleoside phosphate catabolic process (BP)	4	18.9	1.7E−02	Chromatin (CC)	5	11.8	6.4E−03
***Glycosome (BP)***	7	5.9	2.1E−02	Nucleic acid binding (MF)	21	2.5	6.9E−03
***Small-molecule metabolic process (CC)***	11	4.0	2.2E−02	Nucleus (CC)	32	1.9	8.1E−03
***Generation of precursor metabolites and energy (BP)***	5	10.1	4.0E−02	Protein folding (BP)	8	6.0	2.9E−02
Regulation of macromolecule metabolic process (BP)	11	3.5	7.8E−02	***Nucleosome (CC)***	4	12.2	3.2E−02
				Chromosomal part (CC)	6	6.0	5.0E−02
				Kinetoplast (CC)	9	3.8	5.7E−02
**R-DEGs**							
***Structural constituent of ribosome (MF)***	87	5.3	5.9E−44	Cytoskeleton (CC)	113	2.5	1.8E−19
***Translation (BP)***	102	3.4	2.0E−30	ATP binding (MF)	107	2.0	1.8E−11
***Ribosome (CC)***	97	3.5	6.9E−30	Nucleoside triphosphatase activity (MF)	72	2.4	1.5E−10
Biosynthetic process (BP)	167	2.2	1.1E−24	Ciliary basal body (CC)	48	3.0	1.7E−10
Protein-containing complex	192	1.9	1.1E−19	Microtubule organizing center (CC)	48	2.9	8.3E−10
Vacuole (CC)	42	3.1	5.9E−10	Microtubule motor activity (MF)	29	4.2	1.4E−09
Oxidoreductase activity (MF)	65	2.3	2.8E−09	Chromosome segregation (BP)	15	6.5	2.1E−07
Mitochondrion (CC)	143	1.5	4.1E−07	Protein binding (MF)	120	1.7	3.7E−07
***Small-molecule metabolic process (BP)***	72	2.0	9.6E−07	Small-molecule binding (MF)	119	1.7	3.9E−07
***Glycosome (CC)***	38	2.5	3.9E−05	DNA helicase activity (MF)	12	7.2	1.2E−06
***Generation of precursor metabolites and energy (BP)***	21	3.3	2.8E−04	DNA repair (BP)	24	3.4	2.9E−05
Glucose metabolic process (BP)	8	6.4	2.9E−03	Cell projection (CC)	130	1.5	8.8E−05
Cytochrome complex (CC)	6	7.5	3.7E−03	Telomere organization (BP)	7	9.5	2.4E−04
Cofactor binding (MF)	30	2.2	1.1E−02	Kinetochore (CC)	10	5.7	3.7E−04
Proteasome complex (CC)	12	3.2	4.4E−02	Axoneme (CC)	40	2.0	3.2E−03
Nucleotide catabolic process (BP)	10	4.0	5.5E−02	DNA recombination (BP)	9	5.4	6.2E−03
NADP binding (MF)	7	5.1	5.9E−02	***Chromosome (CC)***	20	2.5	2.0E−02
***RNA binding (MF)***	69	1.5	7.3E−02	***DNA replication (BP)***	15	2.8	8.5E−02
**P-DEGs**							
***Translation (BP)***	22	3.22	3.6E−04	***Nucleosome (CC)***	12	7.3	3.7E−06
***Structural constituent of ribosome (MF)***	14	3.65	5.3E−03	DNA packaging (BP)	9	7.8	3.9E−04
***Ribosome (CC)***	18	2.81	1.2E−02	Small-molecule metabolic process (BP)	41	2.2	9.2E−04
Calmodulin binding (MF)	4	15	1.9E−02	Mitochondrion (CC)	78	1.6	1.1E−03
Translation elongation factor activity (MF)	7	6.11	2.5E−02	Chromatin assembly (BP)	7	9.6	1.2E−03
Entry into host cell (BP)	3	28.13	2.8E−02	Ciliary transition zone (CC)	14	3.9	2.3E−03
Nucleic acid binding (MF)	34	1.86	4.7E−02	Oxidoreductase activity (MF)	33	2.3	2.3E−03
***RNA binding (MF)***	23	2.18	6.0E−02	Nucleotide metabolic process (BP)	19	3.2	3.5E−03
				Ciliary plasm (CC)	67	1.7	4.4E−03
				Coenzyme binding (MF)	16	3.1	1.4E−02
				Voltage-gated channel activity (MF)	5	10.3	1.5E−02
				Cofactor binding (MF)	15	2.9	3.7E−02
				***Chromosome (CC)***	15	2.9	4.7E−02

a The top significantly enriched (adjusted *P* values < 0.01) terms are presented. The number of genes, the fold enrichment (FE), and the Bonferroni -adjusted *P* values (*P*-val) are indicated. Term redundancy was manually curated. The type of ontological term is presented next to the term name in parentheses as follows: MF, molecular function; BP, biological process; CC, cellular component. Repeated GO terms on the same cell cycle phase (bold italic) or across stages (gray shading) are highlighted.

10.1128/mSphere.00366-21.9 TABLE S3 Full set of ontology terms for the DEGs in each data set. Download Table S3, XLSX file, 0.2 MB. Copyright © 2021 Chávez et al. 2021 Chávez et al. https://creativecommons.org/licenses/by/4.0/ This content is distributed under the terms of the Creative Commons Attribution 4.0 International license.

The upregulation of ribosomal proteins and genes related to translation regulation in G_1_ arises among the top biological features, being mainly observed in the translatome but also in the proteome. It is worth noting that no G_1_ upregulation of ribosomal protein genes was observed at the transcriptome. A similar translational regulation of ribosomal proteins was observed through ribosome profiling of epimastigote transition to the quiescent metacyclic trypomastigote (23), where the ribosomal proteins are more efficiently translated in the noninfective replicative epimastigote.

Cellular functions related to carbohydrate metabolism and energy production were found overrepresented in G_1_ as for the translatome and transcriptome. This is expected since cell growth mainly occurs at this stage (52, 53). However, these processes were not as clearly overrepresented in the G_1_ proteomic data. This finding suggests that while the synthesis of these enzymes slows down in S phase, their half-life is long enough to maintain the steady-state levels at least at the sampling time. Another remarkable molecular function upregulated in G_1_ is RNA binding, which is modulated at both the translatome and the proteome data sets.

As expected, molecular pathways related to DNA metabolism are overrepresented in S phase. Although these terms are enriched at the three levels, slightly different ones emerged from the different sets of genes. DNA replication was specifically observed in the transcriptome and the translatome, while DNA repair and recombination pathways are observed only for the translatome genes. Meanwhile, the terms observed in the proteome are biased toward DNA packaging and chromatin assembly, perhaps because of the high expression of these genes and the smaller size of the proteomic data set.

Interestingly, terms related to the mitochondrion and the respiratory chain upregulated in G_1_ at the translatome level are also upregulated in S phase at the proteome, suggesting that these mRNAs are loaded onto the polyribosomal compartment in G_1_, thus provoking increased protein levels at S phase.

Finally, cellular functions related to the mitotic spindle formation and organelle organization are overrepresented in the translatome data set at S phase but not yet in the proteome. Coincidentally, these biological functions peaked at G_2_/M in our previous transcriptomic study (44); thus, these proteins may increase their abundance later at G_2_/M phase.

### Analysis of the expression profiles of putative cell cycle regulators.

**FIG 5:**
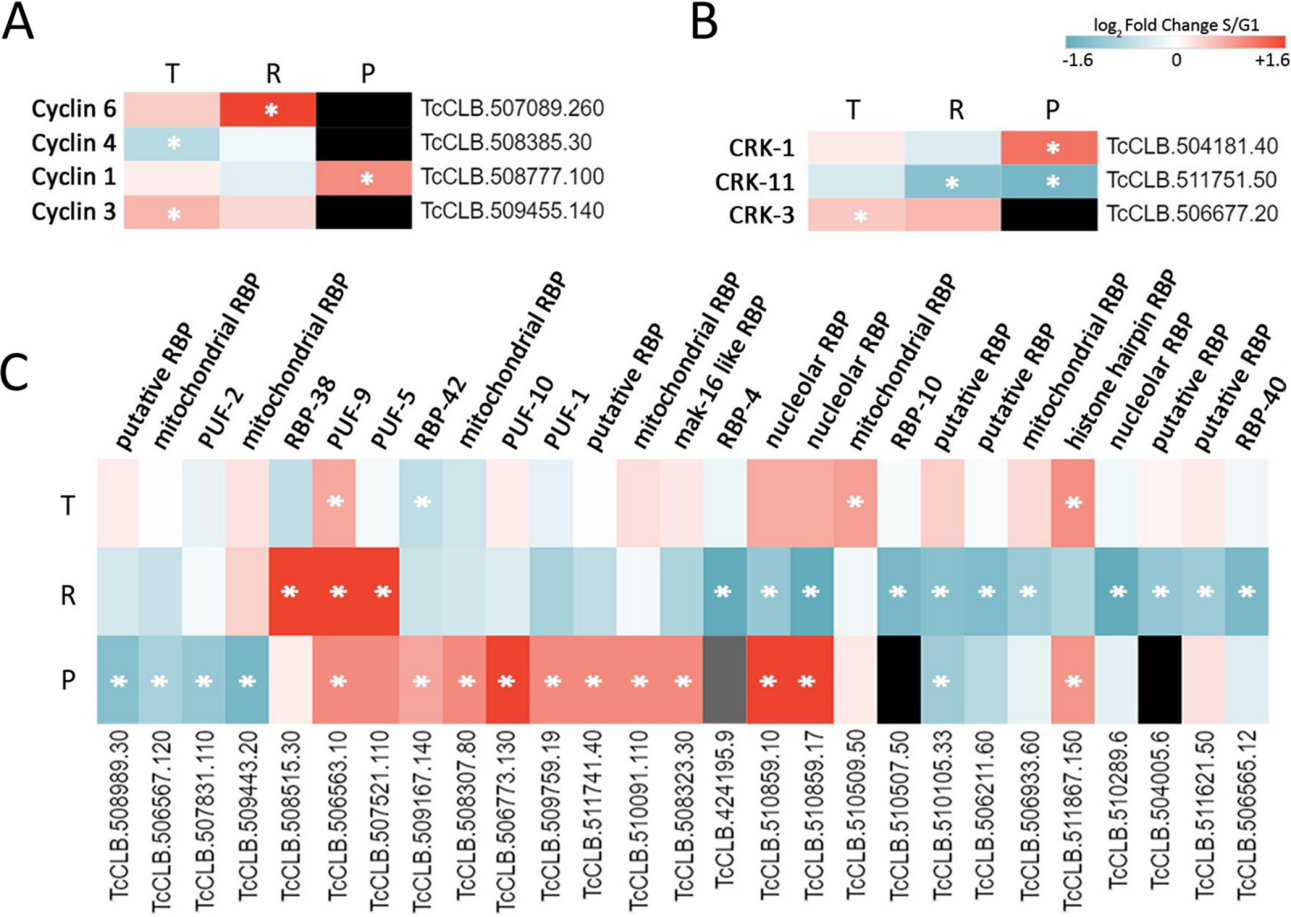
Expression profiles of selected cell cycle regulators. Heatmaps for transcriptome-translatome-proteome (T-R-P) log_2_ fold change of selected differentially expressed genes. (A) Genes coding for cyclins. (B) Genes coding for cdc2-related kinases (CRKs). (C) Genes coding for RNA binding proteins (RBPs). The white asterisk denotes the data set that displayed regulation. Gray shading implies fold change could not be calculated from the proteomic data. Black shading indicates that no protein was detected in any of the replicates.

The 11 T. cruzi cyclins are detected on the translatome data, while only cyclin 6 (TcCYC6, TcCLB.507089.260) is translationally upregulated in S phase. This putative mitotic cyclin was previously characterized by Di Renzo and collaborators, who reported an alteration of G_1_/S transition in TcCYC6-HA (hemagglutinin)-overexpressing parasite populations (30), suggestive of its regulation along the cell cycle. The modulation of TcCYC6 levels evidenced by our study confirms their speculation. In addition, we detect protein levels for only 4 of the cyclins, but only TcCYC1 (TcCLB.508777.100) is significantly modulated, showing an upregulation at the proteome in S phase (Fig. 5A). Unfortunately, there are no further data on the literature for TcCYC1. In addition, no TcCYC4 or TcCYC3 peptides were detected; thus, we were unable to evaluate the effect of the mRNA changes in protein abundance.

The eight annotated trypanosomatid CDK analogs (cdc2-related kinases [CRKs]) are identified in the translatome, and all but one (CRK3) at the proteome. Most of them remain unchanged in G_1_/S, as expected since their functionality is posttranslationally regulated by phosphorylation (46). Interestingly, CRK11 (TcCLB.511751.50) showed concordant downregulation in S phase at both translation and protein levels, while CRK1 (TcCLB.504181.40) is only significantly upregulated at the protein level in S phase (Fig. 5B). While there are no reports for CRK11 in the literature, CRK1 is a deeply studied regulator of the cell cycle in trypanosomatids. CYC2, CYC4, CYC5, three putative G_1_ cyclins, are confirmed partners of CRK1 in T. cruzi (31), whereas the T. brucei ortholog of CRK1 is essential for cell proliferation, promotion of G_1_/S transition, and global translation through phosphorylation of eIF4E4 and PABP1 (54). Other kinases involved in the cell cycle are Polo-like kinase (PLK, TcCLB.506513.160) and Aurora B kinase (AUK1, TcCLB.503799.4). Both are upregulated in S phase, with a 2-fold and 18-fold increase in the translatome for PLK and AUK1, respectively, the latter being one of the top translationally regulated genes. Although the sensitivity of the proteome prevents their reliable quantification, they are detected only in S phase (Table S2). These kinases have a central role in the flagellum duplication and its correct segregation in G_2_ phase (55, 56). Accordingly, both peaked at the G_2_/M phase in our previous transcriptomic analysis (44); thus, they are likely to further increase their protein abundance toward G_2_ phase.

In the context of gene expression regulation in trypanosomatids, RNA binding proteins (RBPs) are candidate surrogates for transcription factors; thus, we searched for the differentially expressed RBPs at the G_1_/S transition. Twenty-eight out of the 87 genes bearing an “RNA-binding” annotation in TriTrypDB (CL Brener Esmeraldo-like haplotype) are differentially expressed in at least one data set. For this set of RBPs, protein levels are frequently independent from ribosome occupancy, suggesting that they are under diverse and complex control mechanisms. RBP40 is the only one that has been characterized in T. cruzi, binding to AG-rich regions in the UTRs of mRNAs coding for transmembrane proteins while undergoing life cycle regulation in both steady-state levels and subcellular localization (57). TcPUF9 is the only RBP showing a significant S-phase upregulation at the three levels analyzed here. This unusual Pumilio family protein stabilizes mRNAs that are upregulated in S phase of T. brucei (58). This finding has been confirmed at the protein level by Benz and Urbaniak, who also identified a cell cycle-modulated phosphorylation site (46). Interestingly, 5 of the 8 Pumilio RBPs annotated in T. cruzi display cell cycle regulation in our data in agreement with their known regulation of cell cycle processes through direct interaction with factors like cyclins, CDKs, PCNA, SLBP, eIF4E, and ribosomal proteins (59). Many of these factors are indeed DEGs in the G_1_/S transition (Table S2).

Cycling sequence binding proteins (CSBPs), associated in two complexes (CSPBI and CSBPII), were described in the closely related trypanosomatid *Crithidia fasciculata* (60) as modulators of mRNA abundance over the cell cycle. CSBPI consists of two zinc finger RNA binding proteins, CSBPA (ZC3H39) and CSBPB (ZC3H40), whose T. brucei orthologs have been shown to bind and repress mRNAs in tethering assays (61). Although their transcripts and protein levels remain unperturbed along the cell cycle (45, 62), TbZC3H39 presents an S-phase-upregulated phosphorylation site (46). Its T. cruzi ortholog, TcZC3H39 (TcCLB.506211.70), has been extensively characterized. It was initially associated with nutritional stress response, binding to cytochrome *c* oxidase and ribosomal protein transcripts and potentially slowing their translation (63). More recently, CRISPR/Cas9 knockout for TcZC3H39 caused major morphological changes accompanied by cell cycle impairment (42), whereas the inability to grow TcZC3H39 null parasites suggests its essential role in cell proliferation. Since this protein is not regulated in the translatome or the proteome in the G_1_/S transition in our T. cruzi study, a posttranslational control, like that of its T. brucei ortholog, could be speculated. In contrast, the unstudied T. cruzi TcZC3H40 ortholog (TcCLB.506211.60) is significantly upregulated in G_1_ in the translatome and possibly in the proteome, indicating different regulatory strategies than in T. brucei.

The CSBPII complex consists of two RBPs with the PSP-1 C-terminal domain (CSBPII_45 and CSBPII_33), whose activities are modulated by phosphorylation. CSBPII_45 binds to a short sequence motif necessary for periodic expression of mRNAs along the cell cycle (64). None of the CSBPII T. brucei orthologs is cell cycle regulated at the protein abundance, although their phosphorylation does change (46). T. cruzi has three orthologs for these RBPs; two of them are identified as CSBPs (TcCLB.508541.190, cell cycle sequence binding phosphoprotein RBP33, and TcCLB.506777.50, cell cycle sequence binding phosphoprotein RBP45) but remain unchanged during the G_1_/S transition at the three levels here analyzed. Meanwhile, the third one is annotated as a hypothetical protein (TcCLB.507611.270), showing a 2.5-fold upregulation in S phase in the translatome and a similar trend in the transcriptome, where it peaks at G_2_/M. Unfortunately, we do not detect this protein in our mass spectrometry data. In agreement, the T. brucei ortholog to TcCLB.507611.270 (PDC3) is upregulated in S phase at protein abundance and also phosphorylation (46).

### Identification of posttranscriptionally coexpressed gene sets in T. cruzi epimastigote G_1_/S cell cycle transition.

**FIG 6:**
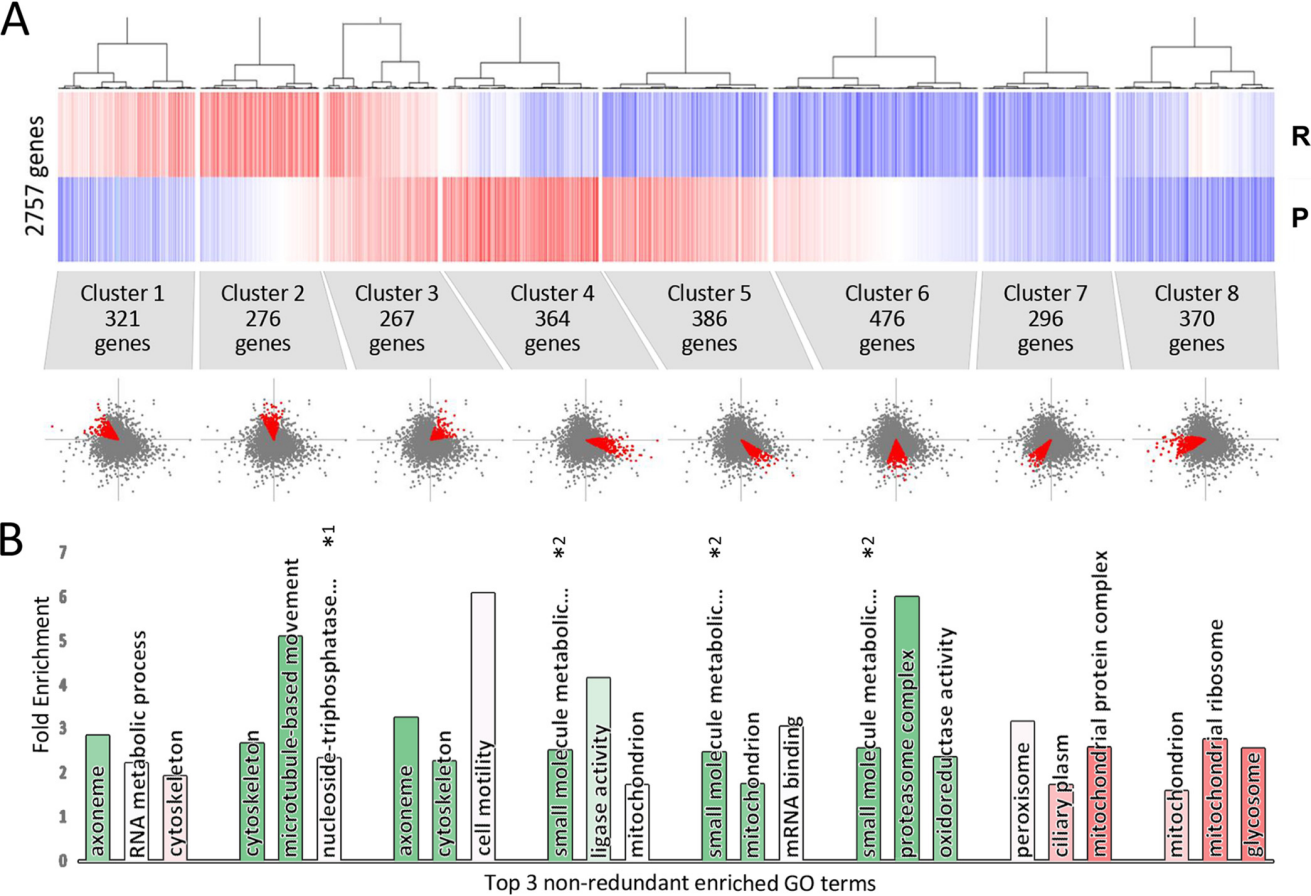
Putative sets of coregulated genes. (A) (Top) Pearson correlation clustering for a set of 2,757 genes with valid fold change values for both the translatome (R) and the proteome (P) data sets. (Middle) The number of genes comprising each cluster. (Bottom) Schematic representation of the location of the genes of each cluster on the fold change diagram presented in Fig. 4C. (B) Top 3 nonredundant gene ontology terms overrepresented in each cluster of genes. Bar heights represent fold enrichment values, and color represents statistical significance (Bonferroni-corrected *P* value) ranging from 3.5E−8 (green) to 7.7E−2 (red). *1, nucleoside triphosphatase activity. *2, small-molecule metabolic process.

To identify putative posttranscriptional regulons operating in the epimastigote G_1_/S transition, we clustered the 2,757 genes with reliable fold change values in both the translatome and the proteome (see Materials and Methods for more details). The Pearson correlation clustering based on the logarithmic S/G_1_ gene fold change resulted in 8 gene groups with similar regulation profiles (Fig. 6A). The top 3 nonredundant Gene Ontology (GO) terms identified for each cluster are listed in Fig. 6B. It is worth noting that the GO analysis presented here is expected to differ from the one in Table 1, since it considers genes with reliable S/G_1_ fold change values in the ribosome profiling and proteomic studies simultaneously. Clusters 2 and 6 are mainly regulated in the translatome without major consequences on protein levels. Cluster 2 is composed of genes upregulated in the translatome at G_1_, which are enriched in terms related to the cytoskeleton and the microtubule motor activity as well as the cilia/flagella. These biological processes point to both the cytoskeletal reorganization that takes place at G_2_/M and the development of the second flagellum that begins at early G_2_ and takes place over this stage before mitosis begins. As these genes raise their ribosome occupancy in S phase but not yet the protein levels, their mRNAs might be loaded on the polyribosome compartment but not yielding increased steady-state protein levels yet. Similar biological processes are enriched in cluster 3, which exhibits an upregulation in both the translatome and the proteome, indicating a regulatory heterogeneity for the proteins involved in these pathways. Then, we looked at the group of genes upregulated in G_1_ phase at the translatome while not changing the protein levels significantly (cluster 6), which are enriched in metabolic processes and energy consumption terms. As mentioned earlier, a high metabolic activity is a hallmark of G_1_ of the cell cycle. Again, a delayed effect of mRNA translation in the proteome due to protein stabilization may explain the lack of corresponding variation of the proteins encoded by these transcripts in G_1_.

Seeking to investigate if the different regulation of each cluster is associated with mRNA and protein metabolism, we calculated the average half-life of both molecules in asynchronous cultures using T. brucei genome-wide mRNA half-life (65) and protein turnover data sets (66). Figure S3 presents box plots of the distribution of logarithmic mRNA and protein half-life of the T. brucei orthologs of the T. cruzi genes identified in each cluster. Only the genes in cluster 1 show a significant reduction of both parameters relative to the average for the orthologous genes identified in the 8 clusters (2,015 mRNAs and 1,673 proteins). Meanwhile, cluster 5 and 6 genes have increased mRNA stability compared to the average. It is worth noting that clusters 1 and 5 present conspicuous antidirectional regulation, suggesting that specific control mechanisms might be needed to ensure proper protein levels for genes undergoing deviated mRNA or protein turnover along the cell cycle.

10.1128/mSphere.00366-21.4 FIG S3 mRNA and protein stability rates per cluster. (A) Schematic representation of the location of the genes from each cluster on the fold change diagram presented in Fig. 4C together with the number of genes representing each cluster. (B) T. brucei genome-wide mRNA decay data from the work of Fadda et al. (65) was analyzed. T. cruzi orthologs were determined for the T. brucei genes with mRNA half-life data, and box plots for these data are presented. The number of genes with data available for each cluster is presented (73% on average). (C) T. brucei genome-wide protein turnover data from the work of Tinti et al., 2019, were analyzed. T. cruzi orthologs were determined for the T. brucei genes with protein turnover data, and box plots for these data are presented. The number of genes with data available for each cluster is presented (61% on average). In both cases Dunn’s multiple-comparison test for non-Gaussian distributed data was performed for each cluster versus the full data set as a control (mean is represented with the dotted line). Significance was determined with and adjusted *P* value lower than 0.05 (**** is <0.0001; *** is between 0.001 and 0.0001; * is between 0.05 and 0.01). Download FIG S3, TIF file, 0.5 MB. Copyright © 2021 Chávez et al. 2021 Chávez et al. https://creativecommons.org/licenses/by/4.0/ This content is distributed under the terms of the Creative Commons Attribution 4.0 International license.

Furthermore, proteins involved in motility represented by GO axoneme and cell motility represented by clusters 1, 2, and 3 are significantly more translated in S phase. This is confirmed at the protein level, more evidently in cluster 3 and for some genes in cluster 2. The apparent uncoupling of translation and protein levels in cluster 1 may be explained by their low mRNA and protein half-life (Fig. S3). This observation suggests that there is a dedicated regulatory mechanism responsible, which opens the possibility of increasing the steady-state protein levels later at S phase. The absence of the ribosomal proteins term enriched (14 genes from Table 1) in the clusters may be due to heterogeneity of the translation and protein abundance dynamic, as these genes were dispersed in 4 different clusters and thus did not lead to ontology term enrichment.

## DISCUSSION

The absence of transcriptional regulation in Trypanosoma cruzi raises the posttranscriptional levels of gene expression control to the main determinant of differential gene expression (16, 21). Although mRNA stability and translatability as well as protein turnover are expected to be key players in gene expression, posttranscriptional regulons remain mostly unknown. Here, we presented the translatome and proteome of G_1_ and S-phase-synchronized T. cruzi epimastigotes, aiming to uncover posttranscriptional mechanisms controlling gene expression at the cell cycle. Since the parasite cell cycle features many characteristics that are divergent from the human host (26, 44, 67), the identification of distinctive characteristics may yield new targets for rational drug design.

Using the most efficient HU synchronization protocol published in the T. cruzi literature (33, 47), we achieved 70% enrichment in G1 and S-phase parasite populations; thus, the differential gene expression will be underestimated due to the presence of 30% undesired cell cycle phases (Fig. 1A and Fig. 2A). In addition, HU treatment, as well as the other cell synchronization protocols, is known to cause effects absent in the natural unperturbed cell cycle; thus, the biological meaning of particular processes should be validated using alternative methods. Yet, the discovery of global gene expression patterns in T. cruzi is not precluded under these conditions, and most of the changes observed are expected to also occur in the unperturbed cell cycle (45). Despite the recognized limitations of the HU synchronization, it is currently the only method that yields an amount of T. cruzi parasites suitable for ribosome profiling (10^9^ parasites per replicate) (68).

Since the epimastigote form is a noninfective stage, our findings would not be directly applicable to the patient treatments but might be useful for parasite-vector control strategies. The decision to study the cell cycle of this parasite form was based on the need for a large number of cell cycle-enriched parasite populations for high-throughput methodologies (68). At the moment, this cannot be achieved working with the intracellular amastigote stage. Nonetheless, the epimastigote form has been widely used as the parasite model to study different biological aspects including sensitivity to chemotherapy drugs. At the same time, molecular mechanisms and machinery of cell replication are likely to be remarkably similar among developmental stages, while the most striking differences would be expected to be at replication control/checkpoint.

Using ribosome profiling and proteomics, we obtained over 31 million sequencing reads and over 500,000 triggered MS/MS spectra, representing 7,248 transcripts and 4,524 protein groups (corresponding to 4,918 genes), respectively. Similar yields were previously reported in trypanosomatid ribosome profiling applying the same cutoff for detection (7,873 transcripts in T. cruzi [23], 7,792 [69] and 7,773 [70] transcripts in T. brucei). Likewise, recent proteomic studies of T. cruzi detected 4,060 (71) and 4,205 (72) protein groups, while a T. brucei cell cycle study detected 4,629 protein groups (46). Therefore, the results of the two omic approaches we carried out to investigate G_1_/S cell cycle phase transition in T. cruzi are consistent with the current state of the art in trypanosomatids regarding sensitivity and coverage. Despite the fractionation of the peptide preparation applied to diminish sample complexity, the lower sensitivity of proteomics relative to ribosome profiling (10, 73) resulted in detected proteins corresponding to medium and highly expressed transcripts. Nonetheless, we identified peptides for 67% of the translated transcripts, while similar studies assessing the translatome and proteome simultaneously in three different human cell lines detected 54% of translated transcripts at the protein level (74).

We found a high correlation among the three data sets and a higher correlation between ribosome occupancy and protein abundance (translatome-proteome) in comparison to transcript levels (both transcriptome-translatome and transcriptome-proteome). This finding agrees with ribosome profiling studies in diverse processes of various model organisms, reviewed by Eastman and collaborators (11). A broader regulation was observed at the G_1_/S transition for the translatome and the proteome levels compared to the transcriptome, seen in the higher number of DEGs (T-DEGs, 305; R-DEGs, 1,784; P-DEGs, 653) as well as in the wider dynamic range of variation observed (see Fig. S2 in the supplemental material), reinforcing the relevance of gene expression control steps occurring after the establishment of mRNA steady-state levels. The slightly larger modulation of the translatome than of the proteome may be influenced by methodological biases; thus, the true relevance of this difference is uncertain. Indeed, transgenomic comparisons are expected to be affected by intrinsic biases of the different techniques. In addition, although the three data sets have been generated using identical and reproducible inter-data-set synchronization protocols and identical subsequent intra-data-set procedures in parallel, we contrasted independent experiments; therefore, batch effects cannot be completely ruled out.

Translational control of specific mRNAs over the cell cycle has been described recently in model organisms employing the ribosome profiling strategy, as reviewed by Aramayo and Polymenis (68). Comparing the numbers of translationally regulated mRNAs in each model, T. cruzi displays a much larger raw number of regulated transcripts and a larger proportion of regulated/detected genes (1,784/7,530, 24%) than both budding yeast (55/3,291, 1.7%) (14) and human cell lines (353/10,841, 3.2%) (12). Moreover, of the 55 transcripts that display differential ribosome occupancy in the budding yeast study, Aramayo and Polymenis (68) suggest that only 17 of these reliably show changes in translational efficiency, once again quite a small number of genes. In this sense, trypanosomatids seem to be far from this scenario, and this is to be expected given that most of the genome is transcribed at a similar rate and thus heavily dependent on posttranscriptional regulation to achieve both differential mRNA and protein levels. Thorough meta-analysis, starting from raw data, would be required to obtain more conclusions from the comparisons of these data sets, since the heterogeneity of methods applied for selection/induction of synchronous cell cycle populations and the nature of such particular gene expression and regulatory mechanisms in trypanosomatids make such comparisons likely unreliable.

A recent proteomic study on T. brucei highlighted the relevance of phosphorylation site abundance, which displayed more widespread changes than protein abundance along 6 time points of the cell cycle obtained by elutriation (46). While we did not study phosphorylation, we were able to identify 408 G_1_/S P-DEGs, a number comparable to the one determined by Benz and Urbaniak (46) (443 protein groups). Despite the methodological differences between the two studies, common terms related to cell cycle are enriched in the regulated proteins, supporting the biological significance of our study.

Both the R-DEGs and the P-DEGs identified in the G_1_/S phase transition are enriched in well-known cell cycle pathways and associated processes. Our analysis reveals that translational regulation magnifies the differences already present at the level of transcript abundance for several biological processes, such as glycosome biology and functions related to energy metabolism for G_1_-upregulated genes. Due to the broader translational control compared to the transcriptome, the R-DEGs not only include but further expand the list of genes observed in the T-DEGs (Fig. S4). In addition, translational regulation provokes a higher magnitude of change than RNA metabolism (Fig. S4). A similar phenomenon is observed for S-phase-upregulated genes, particularly for DNA replication pathways; however, in this case, a different set of genes is involved in the transcriptome and translatome (Fig. S5). Nevertheless, translational regulation is mostly acting on genes that are not regulated at the mRNA level, many of which were not previously studied during the trypanosomatid cell cycle. As an example, translational regulation was observed in G_1_-upregulated genes such as ribosomal proteins and other genes involved in translation, proteasome, and mitochondrial and oxidative processes; likewise, S-phase translationally upregulated genes include microtubule biology-, cytoskeleton-, and motility-related genes and the kinetochore complex. Interestingly, our study found certain groups of translationally regulated genes, such as ribosomal proteins, that have also been identified as translationally regulated in the parasite life cycle (23). This suggests that certain gene sets might be mainly regulated at specific gene expression levels regardless of the biological process studied. The comparison of the translatome and the proteome shows that the majority of the genes regulated are related to different cell processes in each data set, although some ontology terms are regulated in both, such as translation-related terms and RNA binding and chromosome genes (Fig. S5). It is interesting that genes related to the G_2_/M-phase processes increase translation levels on S phase without change in protein abundance. Since it is generally accepted that global translation decreases toward the G_2_/M phase of the cell cycle (12, 75), it is tempting to propose that G_2_/M-related proteins need to be produced in S phase while the translation machinery is still highly active but might be accumulated or stabilized in later G_2_/M phase. Additional time points would be required to test this hypothesis. Overall, the results suggest that the differential gene expression of related gene terms is achieved in multiple ways to finely define the time coordination of biological processes along the cell cycle.

10.1128/mSphere.00366-21.5 FIG S4 Evaluation of transcriptome and translatome repeated GO terms. (A) Venn diagrams for genes comprising cell cycle-overrepresented GO terms in transcriptome (violet) and translatome (red). (B) Scatter column plot for the G_1_ to S-phase transcriptome (violet) and translatome (red) fold change values for the groups of genes resulting from the Venn diagrams (Only T, exclusively transcriptome genes; Only R, exclusively translatome genes; T∩R, genes present at both studies). The number of genes is presented on top of each column. GoPMaE, generation of precursor metabolites and energy; SMMP, small-molecule metabolic process. Download FIG S4, TIF file, 0.5 MB. Copyright © 2021 Chávez et al. 2021 Chávez et al. https://creativecommons.org/licenses/by/4.0/ This content is distributed under the terms of the Creative Commons Attribution 4.0 International license.

10.1128/mSphere.00366-21.6 FIG S5 Evaluation of proteome and translatome repeated GO terms. (A) Venn diagrams for genes comprising cell cycle-overrepresented GO terms in proteome (P, green) and translatome (R, red). SCoR, structural component of ribosomes. Download FIG S5, TIF file, 0.3 MB. Copyright © 2021 Chávez et al. 2021 Chávez et al. https://creativecommons.org/licenses/by/4.0/ This content is distributed under the terms of the Creative Commons Attribution 4.0 International license.

Due to the importance of the identification of T. cruzi cell cycle regulators, we investigated the modulation of cyclins, cyclin-dependent kinases, RBPs, and known cell cycle kinases. Globally, only TcCYC6 is translationally regulated while only TcCYC1 protein level changes (out of the 4 detected), suggesting that the control of cyclins might be mostly posttranslational. As expected, our data indicate that CRKs are probably regulated by posttranslational modifications, but CRK1, CRK3, and CRK11 are controlled at least partially in our data sets. In addition, we found 5 Pumilio proteins that are regulated at the translatome or the proteome during the G_1_/S cell cycle transition, including the ortholog of the well-characterized T. brucei Puf9 (58). Finally, some of the T. cruzi orthologs of the components of CSBP complexes are identified at the translatome and 2 of them undergo translational regulation at G_1_/S not reported in other trypanosomatids, whose direction goes in agreement with prior knowledge of their expression. It is worth noting that our findings invariably support the existing knowledge on the putative cell cycle regulators of trypanosomatids (TcCYC6, CRK1, PLK1, AUK1, PUF9, TcZC3H40, TcCLB.507611.270 [PDC3]), which reinforces the robustness of our omic approach and the usefulness of the translatome analysis for the study of low-expressed genes. In addition, we provide novel support for unstudied candidate T. cruzi TcCYC1; CRK11; PUF1, -2, -5, and -10; and CSPBII proteins.

In this study, we also aimed to identify putative posttranscriptional regulons operating in the epimastigote G_1_/S transition; thus, we focused on the coexpressed genes. Based on the expression at the translatome and the proteome, eight gene clusters with similar expression profiles were identified, changing at a single (only translatome and only proteome) or both levels. The comparison of the concordance of the changes in the translatome and proteome shows both clusters that include genes regulated in the same direction and genes regulated in opposite directions. The regulatory complexity revealed by this analysis suggests that diverse coordinated mechanisms may be needed to define the precise level of specific groups of proteins at different time points during the cell cycle.

A proteome and phosphoproteome study of the T. cruzi cell cycle was recently published (76). In agreement with our findings, the authors observe a similar modulation of ribosomal proteins, oxidoreductase activity, and metabolic pathways. Further comparisons with our findings are precluded by empirical and data analysis differences; thus, a more thorough reanalysis of the data would be required to draw significant conclusions.

In conclusion, we had generated a comprehensive data set uncovering three levels of gene expression, a comparison that has not been assessed in trypanosomatids before. Indeed, very few similar studies are currently published in the literature; thus, further investigation is still required to understand the complexity of the regulation. Our study reveals a larger translational regulation during the G_1_/S transition of the T. cruzi cell cycle in comparison to human and yeast (12, 14) and discloses the translational control of key cell cycle regulators, supporting the importance of translation for gene expression regulation in T. cruzi. In addition, we identified gene groups coregulated at specific levels whose regulatory networks need to be further studied in order to define posttranscriptional gene regulons and their controlling mechanism. Finally, we provide a novel reference data set available for gene-specific as well as systems biology interrogations.

## MATERIALS AND METHODS

### Parasites.

Trypanosoma cruzi epimastigotes forms from the TcI lineage were grown at 28°C in brain heart tryptose (BHT) medium supplemented with 10% heat-inactivated fetal bovine serum (FBS; Capricorn Scientific GmbH). BHT medium was made with 33 g/liter brain heart infusion broth (BHI; Oxoid), 3 g/liter tryptose (Sigma), 0.4 g/liter KCl, 0.3 g/liter glucose, and 3.2 g/liter Na_2_HPO_4_.

### Hydroxyurea-induced synchronization and flow cytometry analysis.

Parasites were synchronized with hydroxyurea (HU) as originally described by Galanti et al. (47) and previously set up for our TcI strain (44). Late G_1_ and mid-S-phase-enriched parasite population samples were collected at 0 and 6 h post-HU release, respectively. A mock sample of parasites was treated under identical conditions except for the use of HU. An aliquot of 2 × 10^6^ parasites/ml was washed twice in cold phosphate-buffered saline (PBS) prior to fixation in 500 μl 70% ethanol in PBS at 4°C for at least 1 h. DNA-specific propidium iodide (PI) staining was conducted by incubation of the fixed parasites for 30 min at 37°C in PBS containing 20 μg/ml PI and 200 μg/ml RNase A. Three technical replicates per biological sample were analyzed for DNA content in a flow cytometer (Accuri C6; BD Biosciences), and the proportions of G_1_, S, and G_2_/M cells in the samples were determined as previously described (77).

### Ribosome profiling and deep sequencing.

Three independent synchronization experiments were prepared in parallel to harvest G_1_- or S-enriched parasite cultures. G_1_-phase and S-phase samples were harvested in independent experiments. Ribosome-protected footprints (RFPs) were generated through nuclease treatment of cell extract in the presence of cycloheximide (CHX) as previously described (10) and recently optimized for T. cruzi by our group (23). Briefly, 2 × 10^9^ cell cycle-enriched parasites were incubated for 15 min at 28°C in 100 μg/ml CHX and washed twice in ice-cold PBS containing CHX at the same concentration. The pellet was resuspended in ice-cold hypotonic lysis buffer (10 mM Tris-HCl, pH 7.5, 10 mM NaCl, 5 mM MgCl_2_, 100 μg/ml CHX, 5 mM β-mercaptoethanol, and cOmplete mini EDTA-free protease inhibitor cocktail by Sigma). Cell lysis was initiated by the addition of NP-40 to a final concentration of 1% and aided by gentle pipetting, with verification by optical microscopy. When complete cell lysis was achieved, it was stopped by the addition of 2 M sucrose to a final concentration of 15%. The postmitochondrial supernatant of the lysate was loaded onto 2 ml of 33% sucrose as the lower cushion and ultracentrifuged for 2 h 45 min at 35,000 rpm on an SW 40 Ti Beckman rotor. Following centrifugation, the RNase protection assay was carried out with Benzonase as the RNase, using 250 units for 10 min at 25°C on the polysome pellet. Treated RNA was extracted (*mir*Vana microRNA [miRNA] isolation kit; Thermo Fisher), and ribosome-protected fragments (approximately 30 nucleotides [nt]) were separated and purified through FlashPAGE electrophoresis as previously described. Library preparations were carried out using the TruSeq RNA library prep kit v2 (Illumina), following the manufacturer’s instructions. The experiments were performed in triplicates, and the RFP libraries were analyzed by deep sequencing on the Illumina Novaseq 6000 (Leidos Biomedical Research, NCI-Frederick, Frederick, MD) to obtain 76-bp single-end reads. The six libraries (triplicates of G_1_ and S-phase ribosome footprints) were prepared in parallel and sequenced in the same sequencer run.

### Sequence read processing, mapping, and differential gene expression.

Read trimming was performed using fastx_clipper (FASTX_Toolkit, v0.0.14) with the parameters *-a* *AAGATCGGAAGAGCACACGTCT* *-c -l 18 -M 10*, in order to retain only reads longer than 18 bp that contained Illumina’s 3′-end adapter with an alignment larger than 10 bp. No further quality filtering was performed after supervision through FastQC analysis showing Phred scores larger than 28 for every base. Reads were mapped to the CL-Brener genome (Esmeraldo-like haplotype, v4.2, downloaded from https://tritrypdb.org/tritrypdb/app) using bowtie2 *–very-sensitive-local* parameters. We choose to work with the Esmeraldo-like haplotype since we obtained a higher mapping percentage with the TcI strain used in our study, compared to the non-Esmeraldo-like haplotype. Reads mapping to mRNA features were counted with HTSeq (v0.6.0) with the default *union* mode. Gene expression is presented as normalized ribosome footprints (nRFPs) resulting from the normalization performed by DESeq2 (78), which accounts for both sequencing depth and transcript length. For global comparisons among the data sets, 15 nRFPs were used as the only cutoff to determine the translatome genes to be included in such analyses. However, genes represented by at least 40 nRFPs were considered for further differential expression analysis as this was found to be the threshold for a stable and low interreplicate index variance (data not shown). Differential gene expression was assessed by the DESeq2 package, and G_1_/S translationally regulated genes were defined as those with a fold change greater than 2 supported by an FDR lower than 0.05. The lists of DEGs were analyzed for enrichment of gene ontology (GO) terms in the online analysis tool available at https://tritrypdb.org/tritrypdb/app, a feature that implements a Fisher exact test on query versus background gene list for overrepresented GO terms. A Bonferroni-adjusted *P* value lower than 0.01 was the cutoff considered for significant overrepresentation. Heatmaps were made on the Broad Institute Morpheus web server using row and column clustering by Pearson correlation (79). Well-translated genes were selected for the periodicity analysis on the ribosomal footprints as recommended by Ingolia et al., 2011 (80). These genes presented a median base coverage throughout the coding DNA sequence (CDS) of at least 10 reads that was calculated with a sliding window of 15 nucleotides, excluding the first 15 and the last five codons. The 5′-end mapping periodicity was calculated with the ribosome-protected footprints mapping on these genes. Periodicity was presented as a plot showing the observed (obs)-to-expected (exp) ratio of the 5′-end footprint mapping distribution in the three different reading frames (81). Additional information on data analysis such as log files and scripts is presented as a zipped folder in Text S1 in the supplemental material.

10.1128/mSphere.00366-21.1 TEXT S1 Set of log files and in-house scripts used during ribosome-profiling data analysis. Download Text S1, TXT file, 0.02 MB. Copyright © 2021 Chávez et al. 2021 Chávez et al. https://creativecommons.org/licenses/by/4.0/ This content is distributed under the terms of the Creative Commons Attribution 4.0 International license.

### Label-free proteomics sample preparation and analysis.

Two independent synchronization experiments were performed in parallel. Intrareplicate G_1_ and S phases were derived from the same synchronized culture. Cell cycle-enriched parasite populations were lysed at 5 × 10^8^ cells/ml in SDS lysis buffer (8% SDS, 200 mM Tris, pH 8.5, 200 mM dithiothreitol [DTT], and cOmplete mini EDTA-free protease inhibitor cocktail by Sigma) at 95°C for 5 min. Peptide samples for analysis by mass spectrometry were prepared as described by Urbaniak and collaborators (82), based on modifications of the filter-aided sample preparation (FASP) procedure (83). Protein samples were defrosted to give a total of 2.5 × 10^9^ lysed cells (0.5 ml), solubilized with 4% SDS, and then reductively alkylated in a 30,000-molecular-weight-cutoff vertical spin filtration unit (Vivascience) using the FASP procedure adapted for the larger volumes used here. The sample was digested with a 1:100 ratio (wt/wt) of trypsin gold (Promega) in the filtration unit for 18 h at 37°C, tryptic peptides were eluted by centrifugation, and the filter was washed sequentially with 1 ml of 50 mM NH_4_HCO_3_ and 1 ml of 0.5 M NaCl. The combined eluent was desalted using a 500-mg C_18_ cartridge (SepPak; Waters) and lyophilized. In order to reduce sample complexity, peptide preparations were fractionated in a high-pH reversed-phase peptide fractionation kit (Thermo Scientific Pierce), increasing the proportion of acetonitrile (ACN) in order to obtain different eluates (flowthrough [FT] = 0% ACN, 2% ACN, 3% ACN, 4% ACN, 6% ACN, 10% ACN, 50% ACN). Later, the eluates were combined into 4 fractions (F1 = FT + 4% ACN; F2 = 2% ACN + 4% ACN; F3 = 3% ACN + 50% ACN, and F4 = 6% ACN) based on peptide quantitation and the hydrophobicity nature of each eluate. Liquid chromatography-tandem mass spectrometry (LC-MS/MS) was performed by the FingerPrints Proteomic Facility at the University of Dundee. Liquid chromatography was performed on a fully automated Ultimate U3000 nano-LC System (Dionex) fitted with a 1- by 5-mm PepMap C_18_ trap column and a 75-μm by 15-cm reverse-phase PepMap C_18_ nanocolumn (LC Packings; Dionex). Samples were loaded in 0.1% formic acid (buffer A) and separated using a binary gradient consisting of buffer A (0.1% formic acid) and buffer B (90% methyl cyanide [MeCN], 0.08% formic acid). Peptides were eluted with a linear gradient from 5% to 40% buffer B over 65 min. The high-performance liquid chromatography (HPLC) system was coupled to an LTQ Orbitrap Velos Pro mass spectrometer (Thermo Scientific) equipped with a Proxeon nanospray ion source. The mass spectrometer was operated in data-dependent mode to perform a survey scan over a range of 335 to 1,800 *m/z* in the Orbitrap analyzer (*R *= 60,000), with each MS scan triggering 15 MS^2^ acquisitions of the 15 most intense ions in the LTQ ion trap. The Orbitrap mass analyzer was internally calibrated on the fly using the lock mass of polydimethylcyclosiloxane at *m/z* 445.120025. The four protein samples (duplicates of G_1_ and S phases) were processed in parallel, and the MS analysis was done in tandem.

### Proteomics data processing.

Data were processed using MaxQuant15 version 1.3.0.5 which incorporates the Andromeda search engine (84). Proteins were identified by searching a protein sequence database containing T. cruzi annotated proteins (version 4.2, downloaded from TriTrypDB, https://tritrypdb.org/tritrypdb/app) supplemented with frequently observed contaminants (porcine trypsin, bovine serum albumins, and mammalian keratins). Search parameters specified an MS tolerance of 6 ppm, an MS/MS tolerance at 0.5 Da, and full trypsin specificity, allowing for up to two missed cleavages. Carbamidomethylation of cysteine was set as a fixed modification, and oxidation of methionines, N-terminal protein acetylation, and *N*-pyroglutamate were allowed as variable modifications. Protein groups were disassembled into individual genes to enable a direct comparison with the sequencing data sets while the multicopy information was retained in the proteomics sheet in Table S2. For detection comparisons among the data sets, at least 1 unique peptide was used as the only cutoff to determine the proteome genes to be included in such analyses. However, for expression comparisons proteins with at least one replicate LFQ value for each cell cycle phase were considered. Only proteins with valid LFQ intensities in the four replicates were analyzed for differential expression, and those displaying a fold change greater than 1.5 and supported by an FDR lower than 0.05 were considered DEGs. A 1.5-fold change was selected as a threshold for proteome DEGs, due to the lower sensitivity and range of the proteomic method. Proteins detected only in one cell cycle stage with LFQ values above the 5th percentile in both replicates were also considered DEGs.

### Clustering analysis.

In order to select a group of genes with reliable fold change values in both the ribosome profiling and the proteomic analysis, we applied a set of filters to the data sets. First, we selected the genes with over 40 nRFPs in the ribosome profiling experiment. Only the proteins classified as “single copy” in Table S2 were considered in this analysis, to avoid redundancy in the expression profiles for proteins of multicopy families. In this case we did not set a probabilistic filter or an arbitrary cutoff in the fold change values, aiming to keep a larger set of genes with data in both studies to build the coexpression profiles. The clustering and heatmaps were obtained from the Broad Institute Morpheus web server using row and column clustering by Pearson correlation (79). An arbitrary cutoff was taken from the observation of the distance matrix that resulted in 8 groups of putative coregulated genes.

### Data availability.

Raw sequences obtained were deposited at SRA in the BioProject PRJNA704643 (https://www.ncbi.nlm.nih.gov/sra/PRJNA704643). The MS/MS raw files were deposited into the Peptide Atlas repository and can be accessed at http://www.peptideatlas.org/PASS/PASS01658.
